# Pathogenicity Induced by Invasive Infection of *Streptococcus dysgalactiae* subsp. *equisimilis* in a Mouse Model of Diabetes

**DOI:** 10.3389/fmicb.2018.02128

**Published:** 2018-09-21

**Authors:** Kohei Ogura, Kayo Okumura, Yukiko Shimizu, Teruo Kirikae, Tohru Miyoshi-Akiyama

**Affiliations:** ^1^Pathogenic Microbe Laboratory, Research Institute, National Center for Global Health and Medicine, Tokyo, Japan; ^2^Advanced Health Care Science Research Unit, Institute for Frontier Science Initiative, Ishikawa, Japan; ^3^Department of Infectious Disease, Research Institute, National Center for Global Health and Medicine, Tokyo, Japan; ^4^Department of Microbiology, Juntendo University School of Medicine, Tokyo, Japan

**Keywords:** *Streptococcus dysgalactiae* subsp. *equisimilis*, diabetes mellitus, db/db mouse, cytokine, lethality, inflammation, interleukin-6, microarray

## Abstract

*Streptococcus dysgalactiae* subsp. equisimilis (SDSE) causes severe invasive diseases such as streptococcal toxic shock syndrome, similar to that caused by *S. pyogenes* (GAS). Invasive SDSE infections are increasing, particularly among patients with diabetes mellitus. Here we investigate the association between the pathogenicity of SDSE and diabetes mellitus in a mouse model, using GAS infection for comparison. Intraperitoneal injection of highly hemolytic SDSE-167 into C57BL6/J mice induced a rapid rise in blood glucose concentrations within 4 h, which was otherwise seen only in mice injected with high doses of hypervirulent GAS mutants. The survival rates of mice injected with SDSE-167 were significantly lower in mice (db/db) with type 2 diabetes than in nondiabetic mice. Injection of db/db mice with SDSE-167 increased the concentrations of cytokines and chemokines, particularly those of interleukin 6 and monocyte chemotactic protein-1. Microarray data indicate that multiple pathways are involved in the pathogenicity of SDSE-167 in db/db mice. These data reveal that the mechanisms underlying streptococcal infection differ between SDSE and GAS.

## Introduction

*Streptococcus dysgalactiae* subsp. *equisimilis* (SDSE), distinguished from *S. dysgalactiae* by Vandamme in 1996, is a β-hemolytic streptococcus that forms large colonies and shows strong β-hemolytic activity on blood agar plates (Vandamme et al., 1996). According to the Lancefield grouping, the SDSE strains comprise mainly groups G and C. SDSE infection causes diseases similar to those caused by *S. pyogenes* (GAS), with the exception of rheumatic fever and rheumatic heart disease (Efstratiou, 1997; O’Loughlin et al., 2007; Broyles et al., 2009).

SDSE is a major cause of invasive β-streptococcal infections in elderly people in Japan (Takahashi et al., 2010). Such infection causes endocarditis, necrotizing fasciitis, and streptococcal toxic shock syndrome (STSS) (McDonald et al., 2007; Brandt and Spellerberg, 2009; Takahashi et al., 2011; Nei et al., 2012; Loubinoux et al., 2013; Tsai et al., 2014). Genomic analysis reveals that SDSE is most closely related to GAS among streptococci and possesses common virulence factors, including streptolysins O and S (SLO and SLS), streptokinase, and M proteins (Shimomura et al., 2011; Suzuki et al., 2011). McNeilly and McMillan (2014) proposed that SDSE and GAS can engage in horizontal gene transfer and recombination. Nikolai et al. reported that invasive SDSE strains exhibit greater cytotoxicity toward keratinocytes and higher SLO activity than noninvasive strains (Siemens et al., 2015).

The number of reports on the pathogenicity of SDSE in humans is increasing. For example, Sikder et al. (2017) found that SDSE induces interleukin (IL-17A)/interferon (IFN)-g-dependent myocarditis and valvulitis, which are hallmarks of acute rheumatic fever and rheumatic heart disease. At least 90% of patients with SDSE bacteremia have other co-morbidities such as malignant tumors and diabetes mellitus (DM) (Woo et al., 2001; Sylvetsky et al., 2002; Cohen-Poradosu et al., 2004; Ekelund et al., 2005; Broyles et al., 2009; Rantala, 2014). DM, hyperglycemia, and impaired glucose tolerance are associated with increased levels of IL-6, tumor necrosis factor (TNF)-α, and C-reactive protein (CRP) (de Rekeneire et al., 2006). Hyperglycemia in patients with sepsis is associated with increased cytokine production and increased mortality (Leonidou et al., 2007). Unfortunately, there is no *in vivo* or *in vitro* mouse model of pathogenesis to study the association between DM complications and SDSE virulence.

To fill this gap in our armamentarium of experimental tools, here we investigate the pathogenicity of SDSE in mice with type II DM (T2DM). SDSE strain 167 (SDSE-167), a strain isolated from patients with invasive infection in Japan. This strain has the highest lethality for mice among our collection of pathogenic strains (Watanabe et al., 2013a). We analyzed lethality of SDSE-167 in T2DM mice and cytokine production in comparison with a GAS strain in order to reveal difference of host responses in invasive infection between SDSE and GAS strains.

## Materials and Methods

### Reagents

Sheep whole blood (citric acid) was purchased from Cosmo Bio (12030205, Japan). Sheep whole blood (Alsevers solution) was purchased from Nippon Bio-Supp. Center (Japan). A High Mobility Group Box 1 (HMGB1) ELISA Assay Kit and Mouse Pentraxin 3/TSG-14 Quantikine ELISA Kit (MPTX30) were purchased from Shino-Test Corporation (Japan) and R&D Systems (Minneapolis, MN, United States), respectively. Alanine aminotransferase (ALT) and albumin were measured using SPOTCHEM D1 (Arkray, Japan). Creatinine was measured using LabAssay Creatinine (FUJIFILM Wako Pure Chemical Corporation, Japan).

### Preparation of Bacteria and Mice

SDSE, GAS, and *Staphylococcus aureus* (SA) strains were cultured in brain heart infusion (BHI) broth overnight at 37°C in an atmosphere containing 5% CO_2_ (**Table 1**). Homozygous (diabetic) and heterozygous (nondiabetic) BKS.Cg-Dock7 m+/+Lepr^db^/J mice, heterozygous B6.BKS(D)-Lepr^db^/J mice, and C57BL/6J mice were purchased from Oriental Yeast Co., Ltd., Jackson Laboratory, and Japan SLC, Inc., respectively. IL-6 knockout mice with a C57BL/6J background were obtained as a gift from Dr. Iwakura (Tokyo University of Science, Japan). All animal experiments were performed in accordance with Act on Welfare and Management of Animals (revised in 2012, Ministry of the Environment, Japan) and approved by The National Center for Global Health and Medicine Animal Experiment Committee.

**Table 1:** Characteristics of strains used in this study.

	Name	Serotype	Isolated From	Isolated in	*emm*
SDSE	167	C	Human	Japan	*STC839.0*
strains	124	G	Human	Japan	*STG480.4* (*STG480.5*)
	117	G	Human	Japan	*STG4974.3*
	168	G	Human	Japan	*STG480.8* (*STG480.0*)
GAS	476	A	Human	Japan	*emm1*
strains	SMD	A	Human	Japan	*emm1*
SA	N315	5	Human	Japan	–

*SDSE, *Streptococcus dysgalactia*e subsp. *equisimilis*; GAS, *Streptococcus pyogenes*; SA, *Staphylococcus aureus*.*

### Hemolysis Assays

Hemolysis assays were conducted according to a previous report with a slight modification (Watanabe et al., 2013a). Briefly, red blood cells were collected from whole sheep blood by centrifugation at 1200 × *g* for 20 min, washed with PBS, and resuspended in PBS containing 0.5% (v/v) (Cosmo Bio 12030205) or 2.0% (Nippon Bio-Supp. Center) bovine serum albumin. After incubation with the SDSE or GAS strains for the indicated times, the supernatant was collected by centrifugation and the absorbance measured at 540 nm. Relative hemolysis was calculated using the absorbance of SDSE-167 samples without 2-mercaptoethanol (SLO enhancer) or trypan blue (SLS inhibitor) as the denominator.

### Injection of Bacteria

SDSE-167 was intraperitoneally (i.p.) injected into mice at the indicated concentrations. To compare the pathogenicity of SDSE and GAS, we also injected mice with GAS-M1-476 (also named M1-d). This strain was isolated from a patient with STSS and has the highest lethality for mice of the agents in our collection (Miyoshi-Akiyama et al., 2003, 2012). These mice were euthanized using sevoflurane (Maruishi Pharmaceutical CO., Ltd.), followed by collection of blood and organs. Glucose concentrations of blood collected from tail veins were measured using NIPRO FreeStyle Freedom Lite (NIPRO, Japan).

### Multiplex Cytokine Assay

Diabetic homozygous (*db/db*) BKS.Cg-Dock7 m+/+Lepr^db^/J and nondiabetic heterozygous (*db/+*) mice were injected i.p. with PBS (control), SDSE-167 (4.5 × 10^6^ colony-forming units [CFU]/mouse), GAS-476 (9.0 × 10^6^ CFU/mouse), or SA (1.0 × 10^7^ CFU/mouse). The mice were euthanized at the indicated times, followed by collection of blood from their hearts. After 30-min to 1-h incubation at room temperature, the blood samples were centrifuged at 1500 × *g* for 20 min. Serum cytokine concentrations were determined using a MILLIPLEX Mouse Cytokine/Chemokine Kit according to the manufacturer’s directions (Merck Millipore).

### Microarray Analysis

Homozygous *db/db* (BKS.Cg-Dock7 m+/+Lepr^db^/J) and heterozygous *db/+* mice were injected i.p. with PBS, SDSE-167 (4.5 × 10^6^ CFU/mouse), or SA (9.0 × 10^6^ CFU/mouse) (*n* = 4 per group). After 8 h, the mice were euthanized, and their livers were stored in RNA *later* (ThermoFisher Scientific). Total RNA was extracted using TRIzol (ThermoFisher Scientific) with an RNeasy Mini Kit (QIAGEN). The RNA samples were sent to Pharma Frontier Co., Ltd. (Japan) for analysis using the Affymetrix GeneChip Mouse Gene ST Array. Raw data (CEL files) were processed using CLC Genomics Workbench (QIAGEN) Gene Analysis. Pathway and gene ontology analyses were performed using iPathwayGuide (Advaita Bioinformatics). Pathway and gene ontology analyses were performed using the KEGG PATHWAY and Gene Ontology Consortium databases (Ashburner et al., 2000; The Gene Ontology Consortium, 2017), respectively. Differentially expressed genes (*p* < 0.05) were identified based on the false discovery rate method (Benjamini and Hochberg, 1995). Genes with ±twofold differences between bacteria-injected and control groups are shown in **Supplementary Tables S1, S2, S4, S5**.

## Results

### The SDSE-167 Strain Had High Hemolytic Activity

**FIGURE 1:**
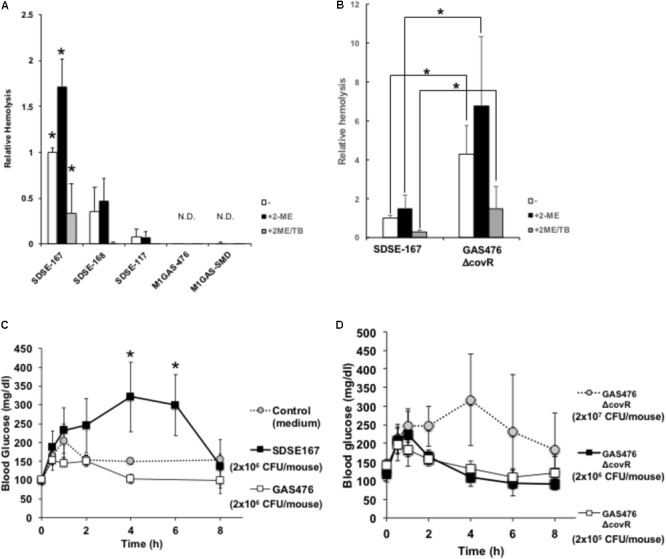
Hemolytic activity of the SDSE and GAS strains. **(A)** A 5% suspension of sheep red blood cells (citric acid, Cosmo Bio) (200 μL) was incubated with 20 mL of a bacterial suspension (optical density at 600 nm, 1) in the presence or absence of 2-mercaptoethanol (2-ME) (50 mM final concentration) (activator of streptolysin O) and trypan blue (TB) (0.01% final concentrations) (inhibitor of streptolysin S) for 3 h. After centrifugation, the absorbance of supernatants was measured at 540 nm. Data represent the mean ± standard deviation (SD) values of three separate triplicate experiments. Asterisks indicate *P* values calculated by ANOVA are <0.01. **(B)** Hemolytic activity of GAS *covR* gene-deleted (Δ*covR*) mutant. A 5% suspension of sheep red blood cells (Alsevers solution, Nippon Bio-Supp. Center) (200 μL) was incubated with 20 mL of a bacterial suspension (SDSE-167 or GAS-476 Δ*covR* mutant) (optical density at 600 nm = 0.05) in the presence or absence of 50 mM 2-ME and 0.01% TB for 3 h. After centrifugation, the absorbance of supernatants was measured at 540 nm. Asterisks indicate *P* values calculated by Student’s *t*-test are <0.01. Data represent the mean ± SD of three separate triplicate experiments. **(C,D)** C57BL6/J mice were injected i.p. with the control (BHI medium), SDSE-167 (2 × 10^6^ CFU/mouse), GAS-476 (2 × 10^6^ colony-forming units [CFU]/mouse) **(C)**, or GAS-476 Δ*covR* mutants (2.0 × 10^5^, 2.0 × 10^6^, or 2.0 × 10^7^ CFU/mouse, respectively) **(D)**. Tail blood glucose concentrations were measured at the indicated times. Data represent the mean ± SD values of two separate quadruplicate experiments (BHI and SDSE-167), a pentaplicate experiment (GAS-476), and a quadruplicate experiment (GAS-476 Δ*covR* mutants) ^∗^*P* < 0.01, ANOVA.

Incubation of a 5% suspension of sheep red blood cells with SDSE-167 for 3 h resulted in significantly greater hemolysis compared with strains SDSE-168, SDSE-117, M1 GAS-476, and M1 GAS-SMD (**Figure 1A**). SLO is inactive in the absence of a reducing agent such as 2-mercaptoethanol (Van Epps and Andersen, 1971). SLS is oxygen-stable but its activity is inhibited by trypan blue (Taketo and Taketo, 1982). The hemolytic activity of SDSE-167 increased in the presence of 2-mercaptoethanol, an SLO activator. This increase was suppressed by additional treatment with trypan blue, an SLS inhibitor. These results suggested that SLO and SLS are expressed at higher levels in SDSE-167 than in other strains. While SDSE-167 had higher hemolytic activity than the other SDSE and GAS strains, the activity was significantly lower than that of *covR* gene-deleted GAS-476 (GAS-476 Δ*covR* mutant), a strain with dramatically elevated hemolytic activity (Heath et al., 1999; Miyoshi-Akiyama et al., 2006) (**Figure 1B**).

### Injection of SDSE-167 Affected Blood Glucose Levels *in vivo*

To determine the effect of SDSE-167 injection on glucose concentrations, SDSE-167 (2.0 × 10^6^ CFU/mouse) and GAS-476 (2.0 × 10^6^ CFU/mouse) were injected i.p., and blood glucose concentrations were measured over time (**Figure 1C**). Blood glucose concentrations increased 4 h after injection to 321 ± 92 mg/dL in SDSE-167-injected mice but not in GAS-476-injected or control (BHI) medium only injected mice. Blood glucose concentrations significantly decreased to 136 ± 76 mg/dL. Injection of the hypervirulent GAS-476 Δ*covR* mutant increased blood glucose concentrations only at the highest dose (2.0 × 10^7^ CFU/mouse) but not at the same dose as SDSE-167 (2.0 × 10^6^ CFU/mouse) (**Figure 1D**). These data suggest that SDSE-167 injection caused a significant increase and a subsequent decrease in glucose concentrations and hemolytic activity *in vivo*.

### Lethality of SDSE-167 Was High in Diabetic Mice

**FIGURE 2:**
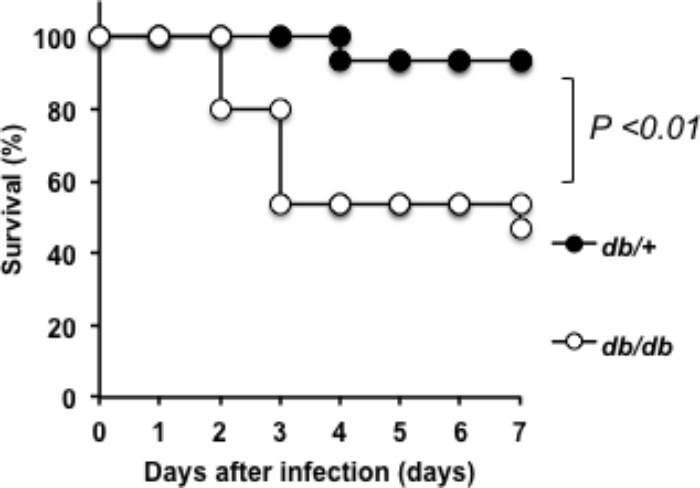
Pathogenicity of SDSE-167 in diabetic mice. SDSE-167 (1.4 × 10^6^ CFU/mouse) was injected i.p. into nondiabetic (heterogeneous *db*/+) and diabetic (homozygous *db/db*) BKS.Cg-Dock7 mice. The figure shows total number of survived mice after three separate pentaplicate experiments (*N* = 15/group) using 5 *db/+* or *db/db* mice for each experiment. The *P* value was calculated using the log-rank test.

We analyzed the pathogenicity of SDSE-167 using a mouse model of DM. First, to determine lethality, survival rates were measured in T2DM (*db/db*) and nondiabetic (heterozygous *db/+*) mice. Kaplan–Meier analysis revealed that 50% of the *db/db* mice died 7 days after injection of SDSE-167 (1.4 × 10^6^ CFU/mouse), while only 1 of 15 nondiabetic mice died (**Figure 2**). The log-rank test showed that the lethality of SDSE-167 was significantly higher in diabetic mice, suggesting that T2DM increases the lethality of SDSE.

### SDSE-167 Induced Production of Cytokines and Chemokines

**FIGURE 3:**
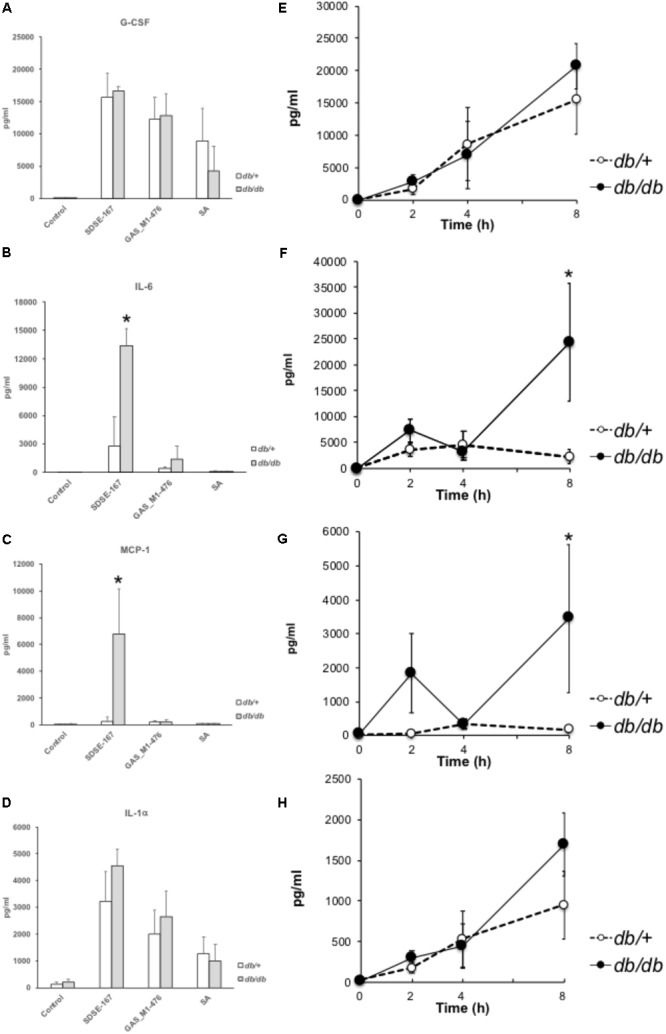
Multiplex cytokines/chemokines assay. **(A–D)** Release of cytokines and chemokines by bacterial injection. Control (medium only), SDSE-167 (4.5 × 10^6^ CFU/mouse), GAS-476 (9.0 × 10^6^ CFU/mouse), or *S. aureus* (SA) strain N315 (1.0 × 10^7^ CFU/mouse) was injected i.p. into nondiabetic (heterozygous *db*/+) and diabetic (homozygous *db/db*) BKS.Cg-Dock7 mice. After 8 h, the mice were euthanized, followed by collection of blood. Serum samples were analyzed using a multiplex cytokine/chemokine assay [**(A)** G-CSF; **(B)** IL-6; **(C)** MCP-1, **(D)** IL-1α]. IL-4, IL-5, IL-7, or TNF-α was not detected using this method. Other data are shown in **Supplementary Figure S3**. Data represent the mean ± SD values from an experiment performed in quadruplicate. Asterisks indicate *P* values calculated by ANOVA are <0.01. **(E–H)** Time course of cytokine/chemokine release. After i.p. injection of SDSE-167 (1 × 10^6^ CFU/mouse), diabetic (*db/db*) and nondiabetic (*db/+*) BKS. Cg-Dock7 mice were euthanized at the indicated times (0, 2, 4, and 8 h), followed by the collection of serum [**(E)** G-CSF; **(F)** IL-6; **(G)** MCP-1; **(H)** IL-1α]. Data represent the mean ± SD values of an experiment performed in triplicate. ^∗^*P* < 0.01, Student’s *t*-test.

We next measured the circulating cytokine concentrations of diabetic and nondiabetic mice early (8 h) after injection (**Figure 3** and **Supplementary Figure S1**). The concentrations of granulocyte-colony stimulating factor (G-CSF) did not differ between *db/+* and *db/db* mice and between SDSE-167 and GAS-476 (**Figure 3A**). Compared with GAS-476-injected mice, SDSE-167-injected mice released higher amounts of IL-6, while IL-6 levels were significantly higher in *db/db* mice (**Figure 3B**). The concentrations of monocyte chemoattractant protein 1 (MCP-1) were significantly higher only in SDSE-167-injected *db/db* mice (**Figure 3C**). The concentrations of granulocyte-macrophage CSF, macrophage inflammatory protein-1, and “regulated on activation, normal T cell expressed and secreted” were significantly higher in SDSE-167-injected *db/db* mice than in GAS-476-injected mice (**Supplementary Figure S1**). The IL-1α level did not differ between *db/+* and *db/db* mice, similar to G-CSF (**Figure 3D**). Injection of *S. aureus* (SA) strain N315 did not induce the release of IL-6, IL-12 (p70), IL-13, or IL-15. As shown in **Figures 3F,G**, the release of IL-6 and MCP-1 increased 4–8 h after injection in *db/db* mice. The concentrations of G-CSF and IL-1α were elevated in both *db/+* and *db/db* mice (**Figures 3E,H**).

### SDSE-167 Induced Release of the Inflammation Markers PTX-3 and HMGB-1

**FIGURE 4:**
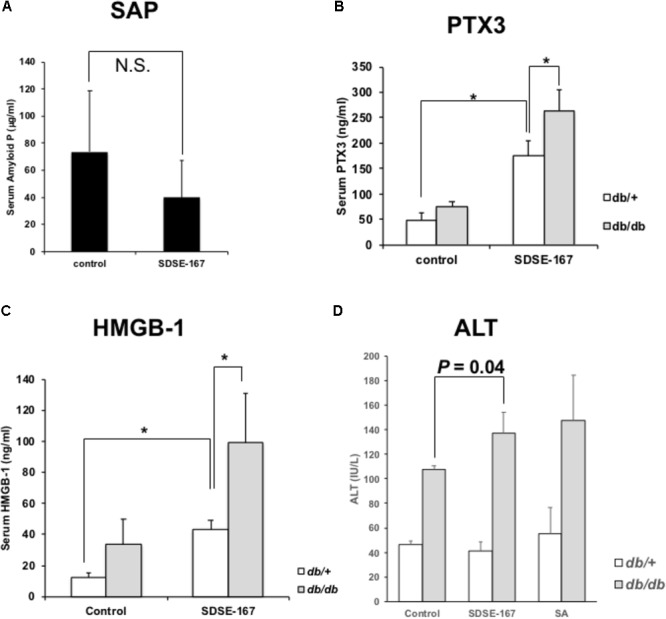
Markers of inflammation. **(A)** Serum amyloid P levels in SDSE-167-injected C57BL6/J mice at a high dose (1.0 × 10^7^ CFU/mouse). PTX-3 **(B)** and HMGB-1 **(C)** levels in the serum of SDSE-167-injected *db/+* or *db/db* C57BL6/J mice (1.5 × 10^6^ CFU/mouse) 6-h after injection. Data represent the mean ± SD values of an experiment performed in quadruplicate. **(D)** Serum alanine transferase (ALT) concentrations in diabetic (*db/db*) and nondiabetic (*db/+*) BKS. Cg-Dock7 mice injected with control (BHI medium), SDSE-167 (2.5 × 10^6^ CFU/mouse), or SA (8.0 × 10^6^ CFU/mouse) for 6 h. Data represent the mean ± SD values of an experiment performed in triplicate. ^∗^*P* < 0.01, Student’s *t*-test.

Because serum amyloid P is homologous to CRP and considered a marker of inflammation, we examined whether the serum amyloid P level was elevated in the SDSE-injected mice. After i.p. injection of SDSE-167 into C57BL6/J (B6) mice, serum amyloid P (a member of the short pentraxin family) was not detectable (**Figure 4A**); however, the concentration of pentraxin-3 (PTX3) (a member of the long pentraxin family) was significantly elevated (**Figure 4B**). PTX-3 concentrations were significantly higher in *db/db* mice. High-mobility group box 1 (HMGB1) was released into the circulation 6 h after SDSE-167 injection (**Figure 4C**). Similar to PTX-3, the concentration of HMGB1 was significantly higher in *db/db* mice, suggesting that SDSE-167 injection induced systemic inflammation in diabetic mice.

To evaluate damage to organs such as the liver and kidney, the serum concentrations of ALT and albumin were measured at 6 h (**Figure 4D** and **Supplementary Figure S2A**). Serum ALT and albumin concentrations did not differ between the control, SDSE-167-injected, and SA-injected nondiabetic mice. In SDSE-167-injected diabetic mice, the ALT and albumin concentrations were slightly higher than those of controls. However, there was no significant difference in ALT or albumin concentrations between mice injected with SDSE-167 and those injected with SA. Serum creatinine concentrations were unchanged after bacterial injection (**Supplementary Figure S2B**). These data indicate that SDSE-167 did not cause severe organ damage during the relatively short time after injection.

### Lethality of GAS and SDSE Was Varied in IL-6 KO Mice

Our multiplex cytokine assay showed that IL-6 levels were particularly elevated in SDSE-167-injected mice. Therefore, we investigated whether IL-6 secretion was required for the lethality of SDSE in mice. IL-6 knockout increased the lethality of GAS476 (**Supplementary Figure S3A**). In contrast, the lethality of SDSE-167 was partially decreased, although the difference was not significant (**Supplementary Figure S3B**). A multiplex cytokine assay revealed that the IL-1 and IL-13 concentrations of wild-type and IL-6 KO mice were unchanged, while other cytokines were changed by IL-6 KO (**Figure 3C**). To determine whether the pathogenicity of SDSE was altered by the inhibition of IL-6 signaling, we tested the effects of an anti-IL6 receptor antibody. We observed that the antibody did not significantly decrease lethality (data not shown).

### Microarray Analysis Indicated the Genes and Pathways Involved in the Pathogenicity

**FIGURE 5:**
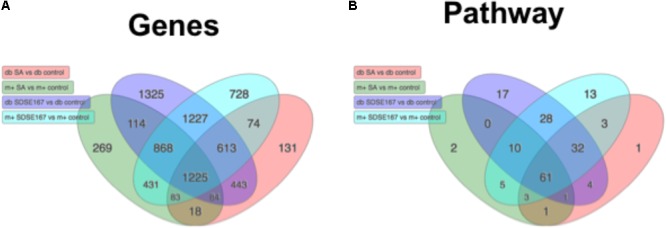
Profiles of gene expression in diabetic mice injected with SDSE strain 167 or SA strain N315. Venn diagram representing **(A)** differentially expressed genes and **(B)** predicted biological pathways. Numbers were obtained from statistical analysis of four groups of control mice injected with PBS as follows: *db/db* (db) BKS.Cg-Dock7 mice injected with SA (red), *db/+* (m+) mice injected with SA strain N315 (green), *db/db* mice injected SDSE strain 167 (purple), *db/+* mice injected with SDSE-167 (blue). The Venn diagrams were drawn using Advaita Bio’s iPathwayGuide (http://www.advaitabio.com/ipathwayguide).

To comprehensively investigate the host response to the bacterial pathogens, we conducted microarray analysis of injected liver tissues. As shown in **Figure 3**, GAS injection induced the release of some cytokines, although the amounts released were less than those induced by SDSE-167 injection. On the other hand, SA injection did not induce cytokine release. To exclude the effect of injection and to identify genes specifically associated with SDSE pathogenicity, we used SA as a control. We observed 1227 differentially genes representing 28 pathways shared between *db/+* and *db/db* mice that were specifically expressed in response to SDSE-167 injection (**Figure 5**). Synthesis of *Ripk2* mRNA increased in SDSE-167-injected *db/+* mice and in SDSE-167-injected *db/db* mice, indicating inflammatory signaling through the nucleotide-binding oligomerization domain-containing protein 1 (NOD)–receptor-interacting serine/threonine-protein kinase 2 (RIPK2) pathway in SDSE-167-injected mice (**Table 2** and **Supplementary Table S1**). The data show that the levels of IL-33, a member of IL-1 family and TNF–α-induced protein 3, an inhibitor of NF-κB, increased in response to SDSE-167 injection. In contrast, SDSE-167 injection significantly decreased the expression levels of genes such as *Per3* and *Pck1* (**Table 2** and **Supplementary Table S1**).

**Table 2:** Top 10 genes with the greatest increase/decrease in expression induced by SDSE-167 injection in *db/+* and *db/db* mice.

Increase in *db/db* and *db/+*			Decrease in *db/db* and *db/+*			Increase in *db/db* specifically		Decrease in *db/db* specifically	
	Log (Fold Change)			Log (Fold Change)			Log (Fold Change)		Log (Fold Change)
Gene	*db/db*	*m+/db*	Gene	*db/db*	*m+/db*	Gene	*db/db*	Gene	*db/db*
Gm10309	10.0	10.0	C630028N24Rik	–8.0	–1.6	Ptgs2	6.8	Gm4794	–3.0
Ripk2	10.0	2.2	Per3	–4.9	–2.4	Il1b	4.5	1600002H07Rik	–2.8
Il33	10.0	1.9	Pck1	–4.6	–1.6	Fgf23	4.4	Arhgef19	–2.7
Rnd1	10.0	3.5	Fmo2	–3.6	–1.4	Il6	3.8	Mtnr1a	–2.7
Tnfaip3	10.0	10.0	Rxrg	–3.5	–1.8	Nos2	3.8	Ptprb	–2.6
Selp	10.0	3.1	Olfml1	–3.2	–2.9	Gbp3	3.7	Sdpr	–2.5
Reg3g	10.0	10.0	Lyst	–3.2	–1.3	Ctla2a	3.4	4732463B04Rik	–2.4
Slc7a6	10.0	4.0	Dbp	–3.2	–3.4	Tmem154	3.3	Smpd3	–2.4
Serpine1	10.0	10.0	Slc46a3	–3.1	–2.5	Clec4e	3.1	Dnajc28	–2.4
Cxcl2	9.2	3.1	Slc16a12	–3.0	–4.1	Car13	2.9	Zmat1	–2.4

*See **Supplementary Tables S1, S2, S4, S5** for details and other genes.*

Pathway analysis indicated that SDSE-167 induced a common response to *Salmonella* injection (**Table 2** and **Supplementary Table S3**). Further, the array data identified 1325 genes representing 17 pathways with altered expression only in SDSE-167-injected *db/db* mice. Prostaglandin–endoperoxide synthase 2, IL-1, fibroblast growth factor-23, IL-6, and nitric oxide synthetase-2 likely contributed to the *db/db*-specific effects of SDSE injection (**Table 2** and **Supplementary Table S4**). Our observation that IL-6 mRNA levels increased in *db/db* mice is consistent with the cytokine assay data (**Figure 3**). Decreased levels of the mRNAs encoded by *gm4794* and *1600002h07rik* were observed, although the functions of these genes are unknown (**Table 2** and **Supplementary Table S5**). The cell cycle and activity of the Th17 cell differentiation pathways were specifically increased in SDSE-167-injected *db/db* mice (**Supplementary Table S6**).

## Discussion

In this study, we found that SDSE-167 has higher hemolytic activity than the other SDSE and GAS strains *in vitro*. SDSE-167 injection caused a significant increase and a subsequent decrease in glucose concentrations and resulted in significantly higher lethality in diabetic mice *in vivo*. In SDSE-167-injected diabetic mice, IL-6, MCP-1, PTX-3, and HMGB-1 concentrations were significantly increased.

Although GAS and SDSE are both β-hemolytic streptococci, SDSE strains caused significantly greater hemolysis than did M1 GAS strains (**Figure 1A**). Invasive infection by SDSE-167 caused a rapid increase in blood glucose concentrations, followed by a decrease (**Figure 1B**). To examine whether these SDSE-167–induced changes in glucose levels were specific to SDSE-167, we conducted comparative analysis using a hyper-virulent *covR*-deleted GAS M1 strain (**Figures 1B,D**). Inactivation or dysfunction of CovR/CovS in GAS significantly increases hemolytic activity (Heath et al., 1999). In a previous report, our group showed in another SDSE strain (strain 124) that a *covS*-deficient mutant induced severe systemic hemolysis in mice (Watanabe et al., 2013b). Despite the presence of an intact *covS* gene in SDSE-167, SDSE-167 produces particularly high SLO and SOS activities. Invasive infection with SDSE-167 caused a rapid increase in blood glucose concentrations followed by a decrease (**Figure 1C**). Injection of GAS-476 did not increase blood glucose levels, even at high doses (4.0–6.0 × 10^6^ CFU/mouse) (data not shown).

Hemolysis generally releases glucose and insulin-degrading enzymes from red blood cells. The release of cytokines in *db/db* mice increased 4–8 h after injection (**Figures 3E–H**), indicating that cytokine production was followed by an increase in glucose concentrations. Sundar et al. (2018) found that the hemolytic activity of GAS correlates with the glucose concentration. However, it is unknown how blood glucose and insulin concentrations are associated with the pathogenicity of SDSE in animal models of DM. Notably, we found that the pathogenicity of SDSE was higher in T2DM model mice than in nondiabetic mice (**Figure 2**). DM is associated with increased susceptibility to injection and sepsis because high levels of glucose inhibit the function of the immune system (Koh et al., 2012). For example, SDSE injection significantly increased the production of IL-1 (α and β) and IL-6 (**Figures 3, 4**); IL-1 is a key mediator of the inflammatory response (Chen et al., 2007).

We show here that i.p. injection of SDSE-167 induced an increase in the concentration of circulating PTX-3 but not serum amyloid P (**Figure 5**). PTX3 is a potent activator of macrophages (Garlanda et al., 2002). In contrast, CRP and serum amyloid P are predominantly synthetized by hepatocytes when stimulated by IL-1 and IL-6; and *Ptx3* transcription can be up-regulated in different cell types in response to diverse stimuli (Casula et al., 2017).

HMGB1 is a ubiquitously expressed nuclear protein secreted by cells of the innate immune system in response to pathogenic agents. This protein is released by injured or dying cells and thus plays a central role in the pathogenesis of infectious inflammation (Andersson and Tracey, 2011). HMGB1 can induce multiple host cell responses, including cytokine synthesis in macrophages, chemotaxis of neutrophils, and proliferation of naive T lymphocytes. HMGB1, which is released during pyroptosis, induces a strong inflammatory response (LaRock and Nizet, 2015). Extracellular HMGB1 is abundant in animals and humans with meningitis caused by *S. pneumonia*e infection, and theHMBG1 levels are associated with the severity of disease in mice and humans (Höhne et al., 2013). HMGB1 levels correlate with T2DM (Wang et al., 2016). Consistent with our results (**Figure 5C**), the concentrations of circulating HMGB1 was higher in mice with T2DM. Tsoyi et al. (2011) found that HMGB1 expression by the macrophage/monocytic mouse cell line RAW264.7 treated with lipopolysaccharide is significantly decreased by metformin, an orally administered drug used to treat patients with T2DM.

The injection of SDSE-167 into mice resulted in increased concentrations of circulating cytokines, particularly IL-6, in diabetic mice (**Figure 3**). IL-6 signaling contributes to the response to injection, inflammation, and the regulation of metabolic processes. IL-6 mediates the host’s defense against pathogens and the survival of GAS-injected mice. In contrast, in SDSE-167-injected mice, IL-6 was associated with lethality, although its influence was not statistically significant (**Figure 3B**). Microarray data show that IL-6 mRNA levels are elevated in SDSE-167-injected *db/db* mice, consistent with the cytokine data (**Figure 3**). IL-6 regulates the activity of the Th17 cell differentiation pathway, which was increased in SDSE-167–injected *db/db* (**Supplementary Table S6**) (Kimura and Kishimoto, 2010).

The identities of inflammatory signaling pathways other than IL-6-dependent pathways that mediate SDSE pathogenicity are unknown. The array data indicate that RIPK2-NOD signaling likely plays a critically important role in pathogenicity. For example, NOD1 and RIPK2 are associated with the autophagy and inflammatory signaling induced by the outer membrane vesicles of bacteria (Irving et al., 2014). Prostaglandin-endoperoxide synthase 2 (COX-2), which is regulated by certain cytokines and growth factors, plays a pivotal role in pathogenicity. Moreover, the induction of COX-2 synthesis by GAS is mediated by cytolysins (Blaschke et al., 2017). These factors may be candidates for further analysis, which is required to gain a comprehensive understanding of the mechanism of SDSE pathogenicity.

In the present study, we analyzed the pathogenicity of SDSE-167 by measuring hemolysis, lethality, cytokine production, and differential gene expression. The scope of our results is limited by the use of only SDSE-167, which is highly lethal in mice. We were not able to conclude whether our findings are applicable to other SDSE strains.

## Author Contributions

KoO performed the experiments, analyzed the data, and drafted the manuscript. KaO performed analysis of lethality for mice. YS contributed to data collection of serum components. TK contributed to collection of bacteria and improved quality of the manuscript. TM-A performed experiments for revision, designed the study, and revised the manuscript for important scholarly content.

## Conflict of Interest Statement

The authors declare that the research was conducted in the absence of any commercial or financial relationships that could be construed as a potential conflict of interest.

## Supplementary Material

FIGURE S1 (Related to **Figure 3**) Multiplex cytokines/chemokines assay. Release of cytokines and chemokines by bacterial injection. Control (medium only), SDSE-167 (4.5 × 10^6^ CFU/mouse), GAS-476 (9.0 × 10^6^ CFU/mouse), or *S. aureus* (SA) strain N315 (1.0 × 10^7^ CFU/mouse) was injected i.p. into nondiabetic (heterozygous db/+) and diabetic (homozygous db/db) BKS.Cg-Dock7 mice. After 8 h, the mice were euthanized, followed by collection of blood. Serum samples were analyzed using a multiplex cytokine/chemokine assay. Data represent the mean ± SD values from an experiment performed in quadruplicate. Asterisks indicate *P*-values calculated by ANOVA are <0.01.

FIGURE S2 (Related to **Figure 4**) Markers of inflammation. Serum albumin and creatinine concentrations in diabetic (db/db) and non-diabetic (db/+) BKS.Cg-Dock7 mice injected with control (BHI medium), SDSE-167 (2.5 × 10^6^ CFU/mouse), or SA (8.0 × 10^6^ CFU/mouse) for 6 h. Data represent the mean ± SD values of an experiment performed in triplicate. Asterisks indicate *P*-value was calculated by Student’s *t*-test.

FIGURE S3 Effects of IL-6 knockout on lethality and cytokine production. Pathogenicities of GAS-476 (6 × 10^6^ CFU/mouse) **(A)** and SDSE-167 (2.5 × 10^6^ CFU/mouse) **(B)** in wild-type (WT) and IL-6 knockout (IL-6 KO) C57BL6/J mice (*n* = 10/group). *P*-values were calculated using the log-rank test. C. Cytokines changed by IL-6 knockout. WT and IL-6 KO mice (*n* = 4/group) were injected with SDSE-167 (4.5 × 10^6^ CFU/mouse). After 6 h, serums were collected for assay. *P*-values were calculated using Student’s *t*-test.

TABLE S1 Genes increased by SDSE-167 injection in both *db/+* and *db/db*mice.

TABLE S2 Genes decreased by SDSE-167 injection in both *db/*+ and *db/db* mice.

TABLE S3 Candidates of pathways induced by SDSE-167 injection in both *db/+* and *db/db* mice.

TABLE S4 Genes increased by SDSE-167 injection specifically in *db/db* mice.

TABLE S5 Genes decreased by SDSE-167 injection specifically in *db/db* mice.

TABLE S6 Candidates of pathways induced by SDSE-167 injection specifically in *db/db* mice.
