# Supplementary material for: Pathogenicity Induced by Invasive Infection of Streptococcus dysgalactiae subsp. equisimilis in a Mouse Model of Diabetes
Source: Front Microbiol. 2018 Sep 21;9:2128. doi: 10.3389/fmicb.2018.02128 (PMC6160533; doi:10.3389/fmicb.2018.02128)
Supplement: FIGURE S1 (Related to Figure 3) — Multiplex cytokines/chemokines assay. Release of cytokines and chemokines by bacterial injection. Control (medium only), SDSE-167 (4.5 × 10^6^ CFU/mouse), GAS-476 (9.0 × 10^6^ CFU/mouse), or S. aureus (SA) strain N315 (1.0 × 10^7^ CFU/mouse) was injected i.p. into nondiabetic (heterozygous db/+) and diabetic (homozygous db/db) BKS.Cg-Dock7 mice. After 8 h, the mice were euthanized, followed by collection of blood. Serum samples were analyzed using a multiplex cytokine/chemokine assay. Data represent the mean ± SD values from an experiment performed in quadruplicate. Asterisks indicate P-values calculated by ANOVA are <0.01. [file Data_Sheet_1.docx]

Supplementary Material

**Pathogenicity induced by invasive infection of *Streptococcus dysgalactiae* subsp. *equisimilis* in a mouse model of diabetes**

Kohei Ogura, Kayo Okumura, Yukiko Shimizu, and Tohru Miyoshi-Akiyama*

*** Correspondence:** takiyam@ri.ncgm.go.jp.

**Figure S1(Related to Figure 3). Multiplex cytokines/chemokines assay**. Release of cytokines and chemokines by bacterial injection. Control (medium only), SDSE-167 (4.5× 10^6^ CFU/mouse), GAS-476 (9.0 × 10^6^ CFU/mouse), or *S. aureus* (SA) strain N315 (1.0 × 10^7^ CFU/mouse) was injected *i.p.* into nondiabetic (heterozygous *db*/+) and diabetic (homozygous *db/db*) BKS.Cg-Dock7 mice. After 8 h, the mice were euthanized, followed by collection of blood. Serum samples were analyzed using a multiplex cytokine/chemokine assay. Data represent the mean ± SD values from an experiment performed in quadruplicate. Asterisks indicate *P* values calculated by ANOVA are <0.01.

**Figure S2 (Related to Figure 4). Markers of inflammation.** Serum albumin and creatinine concentrations in diabetic (*db/db*) and nondiabetic (*db/+*) BKS.Cg-Dock7 mice injected with control (BHI medium), SDSE-167 (2.5 × 10^6^ CFU/mouse), or SA (8.0 × 10^6^ CFU/mouse) for 6 h. Data represent the mean ± SD values of an experiment performed in triplicate. Asterisks indicate *P* value was calculated by Student’s t-test.

**Figure S3. Effects of IL-6 knockout on lethality and cytokine production.** Pathogenicities of GAS-476 (6 × 10^6^ CFU/mouse) (*A*) and SDSE-167 (2.5 × 10^6^ CFU/mouse) (*B*) in wild-type (WT) and IL-6 knockout (IL-6 KO) C57BL6/J mice (n = 10/group). *P* values were calculated using the log-rank test. *C*. Cytokines changed by IL-6 knockout. WT and IL-6 KO mice (n=4/group) were injected with SDSE-167 (4.5× 10^6^ CFU/mouse). After 6 h, serums were collected for assay. *P* values were calculated using Student’s t-test.
